# Creation of chimeric human/rabbit APOBEC1 with HIV-1 restriction and DNA mutation activities

**DOI:** 10.1038/srep19035

**Published:** 2016-01-07

**Authors:** Terumasa Ikeda, Eugene Boon Beng Ong, Nobumoto Watanabe, Nobuo Sakaguchi, Kazuhiko Maeda, Atsushi Koito

**Affiliations:** 1 Department of Retrovirology and Self-Defense, Faculty of Life Sciences, Kumamoto University, Kumamoto 860-8556, Japan; 2 Institute for Research in Molecular Medicine, Universiti Sains Malaysia, 11800 Penang, Malaysia; 3 Bio-Active Compounds Discovery Research Unit, Chemical Biology Research Group, RIKEN Center for Sustainable Resource Science, 2-1, Hirosawa, Wako, Saitama 351-0198, Japan; 4 World Premier International Research Center Initiative, Immunology Frontier Research Center, Osaka University, 3-1 Yamada-oka, Suita 565-0871, Japan; 5 Laboratory of Host Defense, Research Institute for Microbial Diseases, Osaka University, 3-1 Yamada-oka, Suita 565-0871, Japan

## Abstract

APOBEC1 (A1) proteins from lagomorphs and rodents have deaminase-dependent restriction activity against HIV-1, whereas human A1 exerts a negligible effect. To investigate these differences in the restriction of HIV-1 by A1 proteins, a series of chimeric proteins combining rabbit and human A1s was constructed. Homology models of the A1s indicated that their activities derive from functional domains that likely act in tandem through a dimeric interface. The C-terminal region containing the leucine-rich motif and the dimerization domains of rabbit A1 is important for its anti-HIV-1 activity. The A1 chimeras with strong anti-HIV-1 activity were incorporated into virions more efficiently than those without anti-HIV-1 activity, and exhibited potent DNA-mutator activity. Therefore, the C-terminal region of rabbit A1 is involved in both its packaging into the HIV-1 virion and its deamination activity against both viral cDNA and genomic RNA. This study identifies the novel molecular mechanism underlying the target specificity of A1.

The AID/APOBEC family is composed of activation-induced cytidine deaminase (AID), *apolipoprotein B* mRNA editing catalytic subunit 1 (APOBEC1, A1), APOBEC2 (A2), APOBEC3 (A3), and APOBEC4 (A4)^1^. AID and A1 clearly catalyse C-to-U deaminase reactions on ssDNA and/or RNA. A1 was originally characterized as the catalytic component of a complex that mediates the editing of *apoB* mRNA, which encodes a protein involved in lipid transport in gastrointestinal tissues^2^,^3^. In contrast, AID is a DNA editing enzyme that initiates class switch recombination and somatic hypermutation at the *immunoglobulin* gene loci of mature B cells^4^,^5^. This DNA editing activity is essential for B cell diversification. Both AID and A2 are thought to be the precursors of the other APOBEC family proteins, because only *AID*- and *A2*-like sequences are encoded in the genomes of non-mammalian vertebrates^6^. Therefore, AID/APOBEC family proteins seem to have functional redundancy as reported that AID restricts LINE-1 retrotransposon^7^,^8^, whereas mouse A3 might have implications for B cell diversification by promoting immunoglobulin somatic hypermutation during retroviral infection^9^.

We have previously reported that A1s from lagomorphs (rabbit) and rodents (mouse, rat, and hamster) inhibit the infectivity of HIV-1 in 293T cell-based assays^10^. These inhibitory activities are not affected by the presence or absence of the HIV-1 Vif protein^10^,^11^,^12^. The potency of these anti-HIV-1 activities was ranked, and rabbit A1 showed the strongest activity. Rabbit A1 was incorporated into the HIV virion more efficiently than the other A1 proteins, which appeared to contribute to its greater activity. A mutation analysis suggested a deaminase-dependent restriction mechanism, and that both HIV-1 genomic RNA and reverse-transcribed viral cDNA are the substrates of this A1-mediated deamination. The proposal that the A1 proteins from a variety of mammalian species control mobile genetic elements is further supported by several recent studies^11^,^12^,^13^,^14^,^15^,^16^,^17^,^18^.

In this study, we investigated rabbit A1 to determine the regions responsible for its HIV-1 restriction and DNA mutation activities. To address this question, we created a series of chimeric molecules and characterized their activities. Interestingly, our results suggest that chimeric human A1 protein with antiretroviral activity might be a plausible and efficient tool for clinical application in the treatment of HIV-1 infection in combination with other host restriction factors through a genome editing approach^19^.

## Results

### Generation of chimeric molecules from human and rabbit A1s

**Figure 1:**
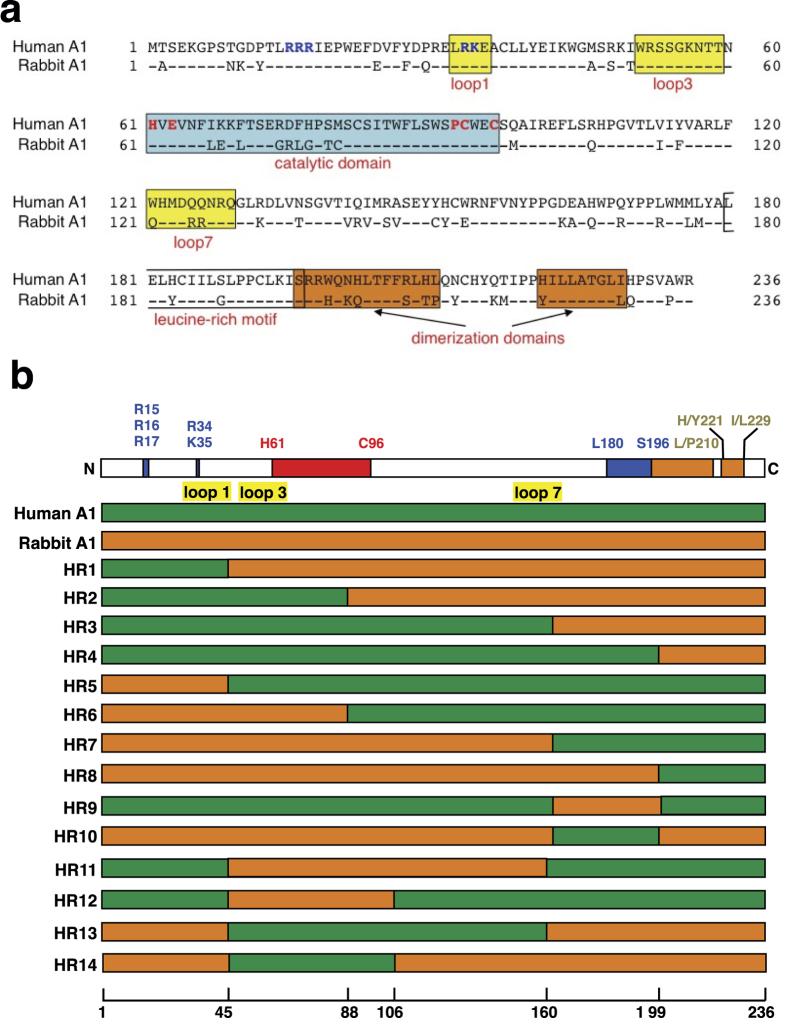
Generation of chimeric molecules from human and rabbit A1. (**a**) A comparison of the predicted amino acid sequences of human and rabbit A1 proteins. Amino acid sequence alignment of A1 from human (GenBank accession number: NM001644) and rabbit (U10695) generated with ClustalW software. The numbers are amino acid residue positions. Identical amino acids are shown with a single dot. The putative bipartite nuclear targeting signals (R^15^, R^16^, R^17^, R^33^, and R^34^) are shown in blue. The H^61^, E^63^, P^92^, C^93^, and C^96^ residues at the catalytic domain (blue box), which play essential roles in catalysis and Zn^2+^-coordination, are indicated in red. The additional loop structures (loops 1, 3, and 7), predicted to form between the α-helices and β-sheet, have been implicated in the proteins’ interactions with their nucleic acid substrates (yellow boxes). The leucine-rich motif (L^180^–S^196^) (clear box) and two dimerization domains (S^196^–L/P^210^and H/Y^221^–I/L^229^) (orange boxes) are indicated. (**b**) Schemes of human A1, rabbit A1, and their derived chimeras. The domain organization of A1 is shown on top of the panel. The green bars correspond to regions from human A1, and the orange bars indicate regions from rabbit A1. The numbers indicate the amino acids of A1 that are replaced in each chimera, and correspond to the A1 amino acids (bottom).

From the studies performed in 293T cells, human A1 shows less anti-HIV-1 activity^10^,^11^, whereas rabbit A1 displays strong restriction activity against HIV-1 through a deaminase-dependent mechanism, acting on both the viral genomic RNA and the reverse-transcribed viral cDNA^10^. Human and rabbit A1s contain 236 amino acids with 75% amino acid sequence identity (Fig. 1a) and contain the conserved active-site core motif (HXE(X)_24–30_PCX_2–4_C) (Fig. 1a, in blue box) in their cytidine deaminase domains (CDs). The N-terminal regions of both A1s contain evolutionarily conserved, basically charged nuclear localization signals (NLSs), which interact with importin α^20^. At the C-terminus, each A1 also has a leucine-rich motif overlapping nuclear export signal (NES) (Fig. 1a, clear box), which associates with CRM1/exportin 1 during the protein’s transport^21^. These sequences contribute to the subcellular localization of A1 in both the cytoplasm and nucleus in cells expressing exogenous A1^21^,^22^. The two dimerization domains at the C-terminal (Fig. 1a, orange boxes) of the A1s are necessary to form the dimer, which is essential for editing *apoB* mRNA^23^,^24^. While preserving these functional domains of the A1 proteins, we generated a series of chimeras combining the human and rabbit A1s (i.e. HR1 to HR14) (Fig. 1b), and examined the regions responsible for the induction of mutations in the HIV-1 genomic RNA and reverse-transcribed viral cDNA. These regions may have utility in an engineered human A1 with anti-HIV-1 activity.

### DNA-mutator activities of chimeric A1s in *E. coli*

**Figure 2:**
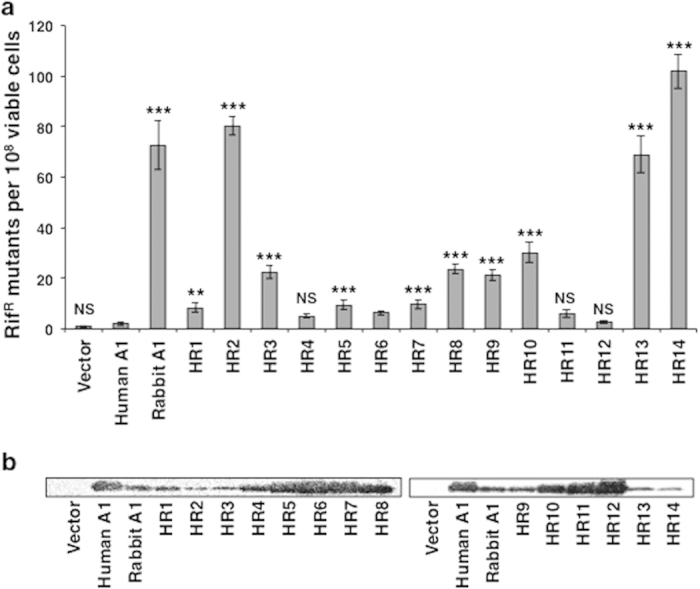
Mutations in *E. coli* genomic DNA caused by A1s and chimeric A1s. (**a**) Abilities of A1s and chimeras to enhance mutagenesis in bacteria. The level of mutagenesis was assessed by plating the bacteria on medium containing rifampicin and counting the number of Rif^R^ colonies. The averages (and standard deviations) of 12 cultures are indicated. *p*-values are shown in comparison to human A1. **p < 0.01, ***p < 0.001, NS, no statistical significance. (**b**) Western blotting analysis of the A1s and chimeras expressed in the bacterial strain, using an anti-HA-tag antibody.

A1 enhances the frequency of mutations in the RNA polymerase beta subunit gene (*rpoB*) of *E. coli*^25^,^26^. Mutations in *rpoB* can be detected by screening for rifampicin-resistant (Rif^R^) colonies. We measured the mutator potential of the chimeric A1 proteins on *rpoB* ssDNA templates by counting Rif^R^ colonies (Fig. 2; data summarized in Table 1). Rabbit A1 markedly enhanced the frequency (>70**-**fold) of Rif^R^ colonies, although human A1 had a negligible effect as a statistically significant difference was not seen in comparison to a vector control (Fig. 2a). The chimeric A1s (HR1-HR14) exhibited various levels of mutator activity (Fig. 2b). HR2, HR13, and HR14 generated remarkably high mutation frequencies, reflected in the generation of Rif^R^ mutants. Curiously, HR13 and HR14, which contained the catalytic domain of human A1, displayed remarkably elevated mutation frequencies (68.9**-**fold and 101.7**-**fold to that of vector control, respectively), nearly as high as that in bacteria expressing rabbit A1. HR3, HR8, and HR9, containing the region that included amino acids 160–199 (Fig. 1b), generated moderate mutation frequencies (21.4**-**fold to 23.7**-**fold), whereas HR10, which lacked the same region, showed a similar mutation frequency, indicating that amino acids 160–199 are not themselves essential for the mutator potential of A1. HR1 induced a lower level of mutation (8.4**-**fold), which was slightly higher than that of human A1. The remaining six chimeras (HR4, HR5, HR6, HR7, HR11, and HR12) showed lower levels of mutation (from 2.6-fold to 9.8**-**fold), although they were present in higher expression levels (Fig. 2b). The expression levels of the chimeras seemed to be inversely related to their ability to generate DNA mutations (Fig. 2). Because the chimeric A1 molecules HR2, HR13, and HR14 displayed strong DNA mutation activity in *E. coli*, this mutator activity must require the C-terminal region of rabbit A1, containing the leucine-rich motif and the dimerization domains.

### C-terminal region of rabbit A1 is partly responsible for its anti-HIV-1 activity

**Figure 3:**
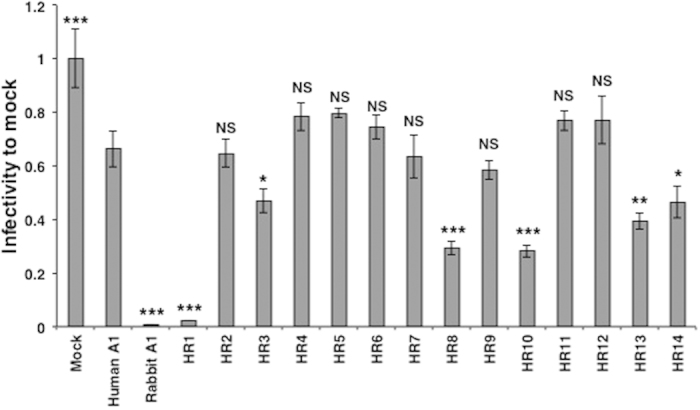
Anti-HIV-1 activity of the A1s and chimeras. A representative single-cycle HIV-1 infection assay in 293T cells. Data are presented as the relative luciferase activity to that detected in cells infected with virions derived from cells that expressed no exogenous APOBEC protein. The average (and standard deviation) of three experiments is indicated. *p*-values are shown in comparison to human A1. *p < 0.05, **p < 0.01, ***p < 0.001, NS, no statistical significance.

We next examined the antiretroviral activity of the chimeric A1s against HIV-1 (Fig. 3, Table 1). Rabbit A1 strongly suppressed viral infectivity after the infection of cells with Vif-proficient HIV-1. The effect of human A1 was not as strong, but it slightly reduced the luciferase activity to 70% of the Mock control, which is consistent with previous observations^10^,^11^,^12^. Compared to human A1, the A1 chimeras, such as HR1, HR3, HR8, HR10, HR13, and HR14, showed statistically significant reductions in infectivity, whereas several A1 chimeras, such as HR5 and HR6, exerted negligible effects. Interestingly, chimeric A1 molecules HR1, HR3, HR13, and HR14, which had anti-HIV-1 activity, contained a large C-terminal sequence. These results suggest that the C-terminal region of rabbit A1, containing one leucine-rich motif and two dimerization domains, as well as its catalytic domain contributes to the protein’s antiretroviral activity.

### Subcellular localization of chimeric A1 molecules

**Figure 4:**
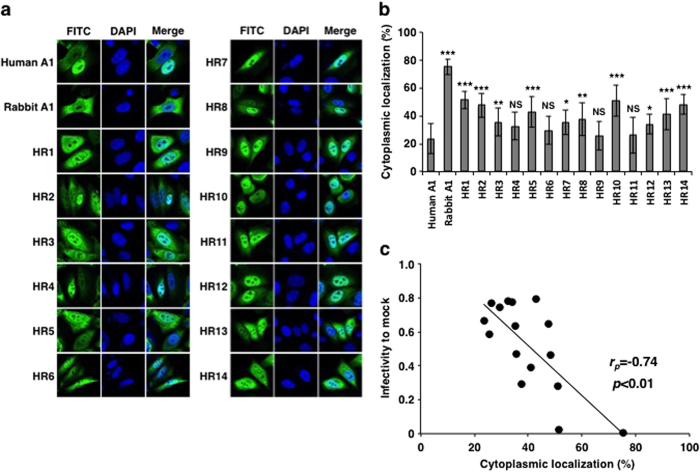
Subcellular localization of the A1s and chimeras. (**a**) HA-tagged A1s and chimeric molecules were expressed in HeLa cells, which were fixed and immunofluorescently stained with an anti-HA-tag antibody and FITC-conjugated goat anti-mouse IgG (green). The images show proteins stained with FITC and then with DAPI to stain the nuclei (blue). (**b**) Cytoplasmic distribution levels of A1s and chimeras. The percentage of cytoplasmic localization of each chimera was calculated by subtraction of fluorescence signal in the nucleus from total fluorescence intensity (n=30). *p*-values are shown in comparison to human A1. *p < 0.05, **p < 0.01, ***p < 0.001, NS, no statistical significance. (**c**) Correlation of cytoplasmic localization levels with HIV-1 infectivity.

To investigate the role of the chimeric A1s in the cell, we examined their subcellular localization. HeLa cells were transiently transfected with expression plasmids encoding the C-terminally HA-tagged A1 chimeric proteins. Previous reports have indicated that a peptide tag at the C-terminus of an A1 protein does not interfere with its function or localization^18^,^22^. Consistent with previous reports, human A1 displayed predominant nuclear localization (76% of total signal was in the nucleus)^21^,^22^, whereas rabbit A1 localized largely in the cytoplasm (75% in the cytoplasm)^18^ (Fig. 4a,b). Most of the chimeric proteins (10 chimeras out of 14) distributed to both nucleus and cytoplasm and their subcellular localizations differed from that of human A1 with a statistically significant difference (Fig. 4a,b). Remarkably, it is demonstrated that the levels of cytoplasmic localization of chimeric proteins statistically correlated with their antiviral activity (Fig. 4c). Therefore, these results suggest that the subcellular localization of chimeras is an important factor for their antiviral activities.

### Chimeric A1s are capable of forming homodimers

The C-terminal region of A1 is reported to be necessary for the formation of homodimers, mediated by protein-protein interactions^23^,^24^. It has also been shown that the RNA-dependent oligomerization of A3G is essential for its packaging into the HIV-1 virion and for the restriction of HIV-1 replication^27^. Because the C-terminal region of A1 includes the dimerization domains (Fig. 1a), we examined whether the chimeric A1s retained their dimerization ability. A3G-HA co-immunoprecipitated with A3G-FLAG and the interaction between these proteins required an RNA bridge because the interaction was significantly impaired by treatment with RNase A, which is consistent with previous studies^27^ (Fig. S1, upper panel, left). A similar requirement for RNA was observed for human and rabbit A1s (Fig. S1, upper panel, middle and right), indicating that the homodimerization of the A1 proteins is also mediated by RNA molecules. This RNA-dependent homodimerization was maintained in all the A1 chimeras (Fig. S1, middle and lower panels). These results indicate that all the chimeras are capable of forming the homodimer, which is a measure of the functional integrity of both A3G and A1 (Table 1).

### Region of rabbit A1 involved in its packaging into HIV-1 virion particle

**Figure 5:**
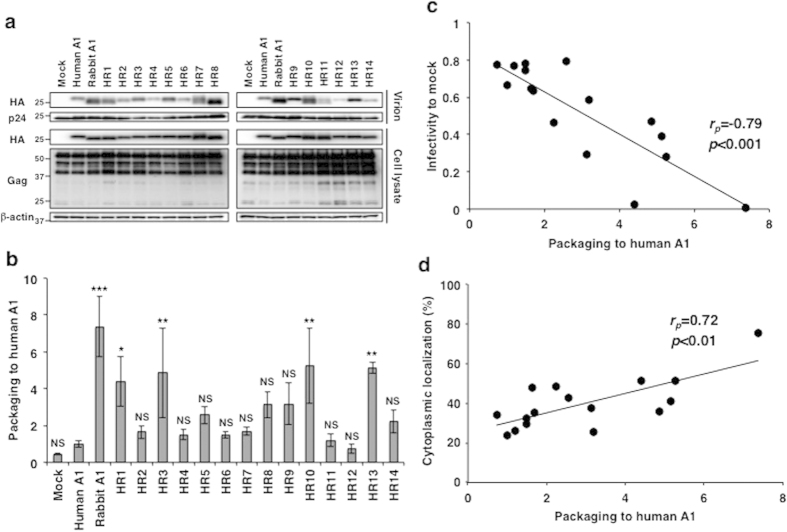
Packaging efficiencies of the A1s and chimeric proteins. (**a**) Cell and virion lysates were subjected to western blotting using antibodies specific for the HA tag and HIV-1 Gag proteins. The blot of the proteins present in the cell lysates was also probed with an anti-β-actin antibody. Data from one experiment representative of three independent experiments are shown. (**b**) Relative packaging efficiency. A1 packaging levels were quantified based on band intensities of HA-tagged A1 normalized to each p24 of the virions and shown graphically as relative packaging to human A1. The average (and standard deviation) of three experiments is indicated. *p*-values are shown in comparison to human A1. *p < 0.05, **p < 0.01, ***p < 0.001, NS, no statistical significance. (**c**) Correlation of packaging levels with HIV-1 infectivity. (**d**) Correlation of packaging levels with cytoplasmic localization.

The packaging of A1 into the HIV-1 virion is an essential step in its deamination of the viral cDNA. We examined whether the chimeric A1s could be packaged into the HIV-1 virion. Rabbit A1 inhibits HIV-1 replication in a DNA and RNA deamination-dependent manner^10^. All the chimeric A1 proteins were detectable at the same levels in the cells infected with Vif-proficient NL-luc virus, and Gag Matrix protein p55 also appeared in each lane (Fig. 5a; in cell lysate). Although the levels of Capsid protein p24 were inconsistent across the cell lysates, it was present at similar levels in the virions (Fig. 5a; p24 in virion). Nevertheless, there were variations in the packaging efficiency of chimeric A1s into the virions (Fig. 5a; HA in virion). The relative packaging efficiency was calculated based on a comparison of the band intensities on a western blot (Fig. 5b). Rabbit A1 was efficiently incorporated into the HIV-1 virions relative to human A1 (~7**-**fold), consistent with previous findings^10^. HR1, HR3, HR10, and HR13 showed reduced packaging efficiency (3 to 5**-**fold) more efficiently than human A1. Although HR8 and HR14 were packaged more efficiently than human A1, there was no statistically significant difference. More importantly, the packaging levels of chimeras into the HIV-1 virions correlated with the levels of cytoplasmic distribution and their antiviral activity (Fig. 5c,d). Therefore, it can be deduced that the C-terminal region of rabbit A1 is involved in its packaging into the HIV-1 virion and the antiviral activity.

### Region of rabbit A1 that edits viral cDNA and genomic RNA

**Figure 6:**
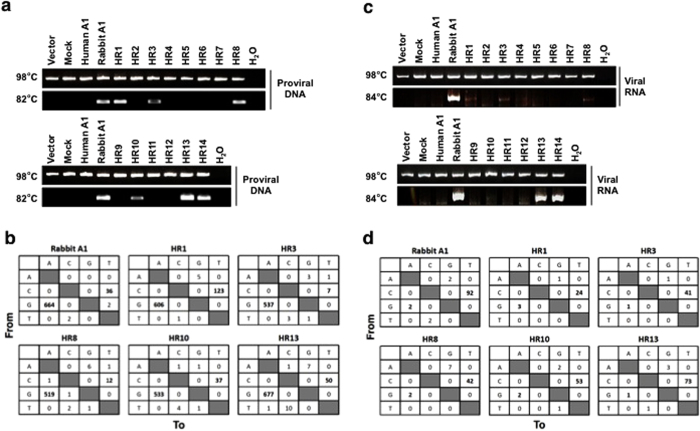
Selective amplification of edited HIV-1 proviral genomes and genomic viral RNA. (**a**) The isolated DNA was amplified using primer sets specific for the *tat/env* region of NL4-3. The nested PCR products denatured at 98 °C were compared with those denatured at 82 °C, after separation with 2% agarose gel electrophoresis. After staining with ethidium bromide, the gel was photographed. (**b**) Mutation matrices of G-to-A and C-to-T mutations in the *tat/env* gene in the proviral DNA of Vif-proficient virus amplified with PCR after denaturation at 82 °C. The boxes show the differences between the nucleotide sequence of the *tat/env* gene and the observed sequence in the presence of rabbit A1 or the indicated chimeric A1 proteins (*n* = 12, 6,384 bp). (**c**) Isolated viral RNA was amplified using primer sets specific for the *tat/env* region of NL4-3. The nested PCR products denatured at 98 °C were compared with those denatured at 84 °C, after separation with 2% agarose gel electrophoresis. After staining with ethidium bromide, the gel was photographed. (**d**) Mutation matrices of G-to-A and C-to-U mutations in the genomic RNA of Vif-proficient virus. PCR was performed with denaturation at 84 °C. The boxes show the differences between the nucleotide sequence of the *tat/env* gene and the observed sequence in the absence of A1 or the presence of human, rabbit, or chimeric A1 protein (*n* = 12, 6,384 bp).

Both the HIV-1 genomic RNA and its viral cDNA are substrates of rabbit A1-mediated deamination^10^. Therefore, we investigated whether the chimeric A1s also targeted DNA and RNA as substrates. HIV-1 genomic RNA and its proviral DNA were recovered from the viral particles and the infected cells, respectively. The recovered proviral DNA and viral genomic RNA were subjected to both conventional PCR and 3D**-**PCR, which allows the selective amplification of AT-rich sequences when the denaturation temperature is reduced^28^. PCR with a denaturation temperature at 98 °C amplified products from the proviral DNAs of Vif-proficient virus, regardless of the presence or absence of A1s (Fig. 6a). However, in the presence of rabbit A1, 3D-PCR generated a DNA band at a denaturation temperature of 82 °C, and the PCR products included greater numbers of G-to-A (664 events) and C-to-T mutations (36 events) in the plus-strand DNA than the products generated at 98 °C (67 and 5 events, respectively) (Fig. 6b and S2). The chimeric A1s which had antiviral activity, such as HR1, HR3, HR8, HR10, HR13, and HR14 showed DNA bands that were amplified by 3D-PCR (Fig. 6a). The sequence analysis confirmed that these chimeras had the high mutation rates in the PCR products with G-to-A (513–803 events) and C-to-T (0–84 events) mutations (Fig. 6b).

Rabbit A1 but not human A1 generated a strong band of mutant PCR product from the viral genomic RNA when the denaturing temperature was reduced to 84 °C using Vif-proficient virus (Fig. 6c), and more C-to-T mutations (92 events) were induced than at 98 °C (34 events) (Fig. 6d and S3). The chimeric A1s showed only slight but consistent bands with their antiviral activity on 3D-PCR (Fig. 6c), with significant C-to-T mutations (HR1, 24 events; HR3, 41 events; HR8, 42 events; HR10, 53 events; HR13, 73 events; and HR14, 59 events (data not shown)). Taken together, these editing assays demonstrate that the chimeric A1s regulate both DNA and RNA editing, and the deaminase activities against DNA and RNA are associated with the antiviral activity (Table 1).

## Discussion

This study demonstrates that the anti-HIV-1 activity of rabbit A1 was significantly influenced by the acquisition of several regions from human A1. Our results show that the C-terminal region, containing one leucine-rich motif and two dimerization domains, is involved in the packaging of A1 into the HIV-1 virion and the deamination of its nucleic acid substrates. However, the transfer of the C-terminus alone from rabbit A1 to human A1 conferred only partial antiviral activity upon the chimeric proteins, which suggests that other factors (e.g., proper folding of the protein, modifications to the protein, involvement of cellular factors) are required for its maximum anti-HIV-1 activity.

**Figure 7:**
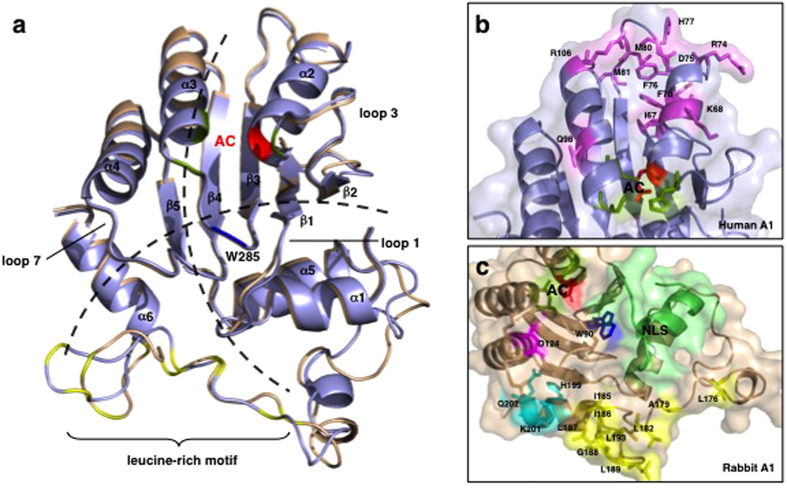
Comparison of the structures of the human and rabbit A1 proteins. (**a**) Homology models of human A1 (light blue) and rabbit A1 (wheat) show structural conservation relative to A3G-CTD (Fig. S1). The ribbon schematics show the core five hydrophobic β-sheets (β1–β5) surrounded by six α-helices (α1–α6). The loop structures (loops 1, 3, and 7), predicted to form between adjacent α-helices and β-sheets, have been implicated in the interaction between the protein and its nucleic acid substrates. The two proposed models of nucleic acid binding to the proteins are represented by dotted lines. In the Zn^2+^-coordinating active core (AC), cysteine (green) and histidine (red) residues are indicated. W^285^ (blue) was reported to be involved in the enzyme’s deamination activity in A3G-CTD and is conserved in human and rabbit A1s. The C-terminal regions of both A1s contain a leucine-rich motif (leucine residues colored yellow). (**b**) Residues in human A1 that differ from those in rabbit A1 in the substituted region of chimera HR14 are shown as sticks (violet). (**c**) In rabbit A1, residue D^124^ (magenta), implicated in the local dinucleotide preference of A3G, is conserved but the residues surrounding it differ from those in human A1. The NLS located in the N-terminal region of the protein is shown in lime green. The leucine-rich motif (Leu and aliphatic residues; yellow) located in the C-terminal region is followed by residues that differ from those in human A1 (H^199^, K^201^, and Q^202^; cyan). Homology models were built with SWISS-MODEL and the images were generated with PyMol.

To glean the results of the assays from a structural perspective, we built homology models (Fig. 7a and S4) to assess the structural elements (such as the NLS, catalytic site, leucine-rich motif, and the dimerization domains) that may contribute to the mutagenic activity of rabbit A1 against ssDNA. Chimera HR14 showed the highest mutagenic activity, and based on the model, the catalytic core section of human A1 (Fig. 7b), which was grafted onto a rabbit A1 scaffold, comprised the structurally conserved α2-β3-α3 active site for the deamination of cytidine (Fig. 7b). More positively charged residues were also found in this region in human A1, which may increase its interaction with the negatively charged DNA backbone, contributing to the mutagenic activity of HR14. The residues surrounding the active site were mainly conserved in both A1s. Although certain domains such as the NLS-containing N-terminal region of rabbit A1 is necessary for mutagenic activity (i.e. HR1), the C-terminal leucine rich motif must also be present (e.g. HR5, HR6 and HR7). The models indicate that the leucine-rich motif region is involved in the formation of a hydrophobic core and in the maintenance of the monomeric structure. Therefore, the stabilization of the hydrophobic core is likely to contribute to the protein’s mutagenic activity because when the leucine-rich motif of rabbit A1 (Fig. 7c) was substituted with that of human A1 (chimera HR10), its activity was reduced by more than half. The model shows that the leucine-rich motif and the dimerization domains are not in close proximity to the catalytic domain. Therefore, they may be part of a potential dimer interface, and work in tandem with the catalytic domain to achieve the antiviral activity of rabbit A1^10^.

The restriction activity of A3G has been studied most extensively because it was the first A3 enzyme to be discovered^29^,^30^. Encapsidated A3G inhibits viral replication in target cells, mainly by catalysing C-to-U deamination in hotspots on the proviral ssDNA substrate. This anti-HIV-1 activity, in a Trojan-horse-type mechanism, has also been demonstrated for A3DE, A3F, and A3H haplotype II^31^,^32^,^33^,^34^. In addition, several studies have demonstrated that A3G restricts viral replication by deaminase-independent mechanisms^35^,^36^,^37^,^38^. Of note, rabbit A1 is efficiently packaged into HIV-1 virions and affects HIV-1 replication strongly by targeting both genomic RNA and reverse-transcribed viral cDNA^10^. Similar to A3G, the antiviral activity of rabbit A1 also includes deaminase-independent mechanisms^10^. In contrast, human A1 slightly inhibits HIV-1 replication without the hallmarks of deamination, regardless of its packaging into HIV-1 virions. The little antiviral activity of human A1 could be attributed to the deaminase-independent mechanisms, and the lack of deaminase activity could explain its limited antiviral activity. Indeed, all the chimeras were packaged into viral particles, and their antiviral activity were at least a level comparable to that of human A1, suggesting that the deaminase-independent mechanisms partly contribute to the restriction activity of chimeras. This slight antiviral activity is enhanced by increasing the packaging into the HIV-1 virion and the deamination of its nucleic acid substrates by the transfer of the C-terminal region of rabbit A1, containing the leucine-rich motif and the dimerization domains. However, it remains unclear how this region regulates its efficient packaging into the HIV-1 virion and its deamination activity on its nucleic acid substrates. Further studies will shed light on the evolutionary history of RNA editing by the A1 deaminases and their ancestral functions, with those of other members of the AID/APOBEC family.

## Methods

### Cell lines and antibodies

293T and HeLa cells were maintained in DMEM supplemented with 10% fetal calf serum (Gibco). An anti-p24 CA antibody has been described previously^39^. Antibodies against HA (C29F4, Cell Signaling Technologies and HA.11, Covance), FLAG (M2, Sigma-Aldrich) and β-actin (AC-74, Sigma-Aldrich) were commercially available.

### Plasmid construction

Plasmids able to express the hemagglutinin (HA)-tagged version of human and rabbit A1s, as well as human A3G, had been previously described^10^,^18^. To create chimeric genes of human and rabbit A1s with HA epitope tag at the C-terminus, two-step PCR method was used. As a first step, 5′ or 3′ PCR fragment was independently obtained by conventional PCR with PrimeSTAR HS DNA polymerase (Takara). As a second step, the overlapping PCR fragments were mixed and then subjected to 2 cycles of the following PCR reaction (98 °C for 10 sec and 68 °C for 1 min) to obtain chimeric genes, followed by conventional PCR reaction to amplify the chimeric product using outside primer set. Fragments encoding HA-tagged human or rabbit A1s in the pCAGGS expression vectors were used as template for most of the constructions^10^ except that HR1 or HR5 were used as a template of 5’ fragments of HR11 and HR12 or HR13 and HR14, and HR8 or HR4 were templates for 3’ fragments of HR9 or HR10. Combination of primer set for the creation of each chimera is shown in Table S1. The amplified chimeric genes were cloned into pCR-Blunt (Invitrogen) vector and sequenced. Then, amplified products were inserted into *Eco* RV and *Not* I sites of pCAGGS expression vector. Regions of FLAG-tagged A1s and chimeras were amplified using primer set (Table S1) and then cloned into *Eco* RV and *Not* I sites of pCAGGS expression vector as described above. Antisense primer encoded the FLAG-epitope sequence DYKDDDDK. To generate the bacterial expression vectors encoding the respective chimeras, fragments of the chimeric genes in the relevant pCAGGS-based plasmid, including the C-terminal HA tag, were PCR-amplified by using oligonucleotides (Table S1). The amplified PCR products were inserted into *Xho* I and *Pst* I sites present in the bacterial expression plasmid pTrcHis A (Invitrogen) as described previously^18^. All constructs were verified by DNA sequencing.

### Bacterial mutator assay

An *E. coli*-based DNA mutation assay was performed by transforming the uracil DNA glycosylase-deficient *E. coli* strain BW310 with the pTrcHis A parental plasmid and vectors encoding the various chimera cDNAs as described previously^18^. Briefly, twenty colonies of the transformed bacteria selected on plates containing ampicillin were cultured in 2 ml of LB medium plus ampicillin and 1 mM IPTG overnight at 37 °C, and then one hundred microliters of the saturated culture was plated on LB plates containing 100 μg/ml of rifampicin. The total number of Rif^R^ colonies per plate was counted 24 h later. For viable cells, appropriate dilution was plated onto LB plate containing ampicillin and mutation frequencies were calculated as Rif^R^ colonies per viable cell. To verify protein expression, 100 μl of the saturated IPTG-induced culture was lysed and analyzed by western blotting as described below.

### Western blot analysis

Cell and virion lysates, and immunoprecipitates were fractionated by SDS-PAGE, transferred to a PVDF membrane (Millipore), and blocked with 4% milk in PBS containing 0.1% Tween 20. Subsequently, membranes were incubated with the primary antibody, biotin-conjugated anti-mouse IgG (Sigma-Aldrich), streptavidin-conjugated horseradish peroxidase (Sigma-Aldrich), and developed using Chemi-Lumi One (Nacalai Tesque). Signals were visualized by a VersaDoc 5000 Imager (Bio-Rad Laboratories).

### Homology modeling and analysis of the A1 structures

Homology models of human and rabbit A1 proteins (UniProt IDs: P41238 and P47855) were built using Swiss-Model server^40^,^41^,^42^ with the C-terminal catalytically active domain (CD2, residues 185–373) of the human A3F crystal structure (PDB: 4IOU) as a template. The built models were visualized and manually aligned to the A3G catalytic domain structure (CD2, residues 197–384) (PDB: 3IR2) using PyMol (The PyMol Molecular Graphics System, Version 0.99rc6, Schrödinger, LLC). Amino acid sequences were aligned using the Clustal Omega Program in UniProtKB^43^. To highlight selective amino acids, corresponding amino acids of the respective proteins were mapped to the built models and colored in PyMol. Figures of the structures were generated using PyMol.

### Immunofluorescence confocal microscopy analysis

2 × 10^4^ HeLa cells were seeded onto 8-well Lab-Tek Chamber Slide (Nalge Nunc International) and immunofluorescence studies were performed at 24 h after transient transfection using FuGENE as previously described^18^. Cells were subsequently fixed with 4% formaldehyde in PBS for 30 min, permeabilized with 0.1% Triton-X100 in PBS for 2 min at room temperature, and subsequently washed 3 times in PBS. Then, the cells were treated with 0.1M glycine/PBS for quenching and 0.3% BSA/PBS for blocking. For APOBECs staining, coverslips were incubated in a humid chamber at 37 °C for 1 h with an anti-HA antibody (HA.11; 1:1000 dilution) in 0.3% BSA/PBS. A FITC-conjugated goat anti-mouse IgG (Sigma-Aldrich; 1:300) in 0.3% BSA/PBS was then added and incubation continued for an additional hour. Subsequently, DAPI staining (Invitrogen; 1 μg/ml) was performed for 5 min. The coverslips were mounted with FLUORESCENT MOUNTING MEDIUM (DAKO). Fluorescence pattern were visualized with a Zeiss LSM 700 laser-scanning confocal microscopy. The images were captured using IPLab and processed using Adobe PhotoShop 4.0 software. Fluorescence intensity was analyzed by using ImageJ software. Fluorescence intensity in the nucleus was measured based on DAPI signal and distribution to the cytoplasm was calculated by subtraction of nuclear intensity from total signal.

### Immunoprecipitation assay

293T cells were cotransfected with 0.5 μg of FLAG- and HA-tagged APOBEC expression vectors, and 24 h later, cells were harvested and then solubilized by lysis buffer (50 mM HEPES pH7.2, 125 mM NaCl, 10% glycerol, 0.1% NP-40 and protease inhibitor cocktail (Sigma-Aldrich)). The cell lysates containing 20 μg (for A3G) and 100 μg (for A1s and chimeras) of total protein were immunoprecipitated by an anti-FLAG antibody bound to Dynabeads^®^ M-280 Sheep anti-mouse IgG (Invitrogen). The precipitated proteins were analyzed by western blotting as described above. To test RNA dependency of dimerization, the cell lysates were treated with RNase A (50 μg/ml, Qiagen) at 37 °C for 1h, before immunoprecipitation. HA-tagged APOBEC protein expression was confirmed by Western blotting with an antibody specific for HA or FLAG epitope tag.

### Viral preparation and infectivity assay

VSV-G pseudotyped HIV-1-based luciferase reporter virus stocks were produced in 293T cells by cotransfection of 1.5 μg of Vif-proficient pNL4-3 Luc E^-^R^−^44, together with 1.0 μg of pVSV-G and 0.5 μg of one of several expression vectors encoding APOBEC proteins which are HA-epitope tagged or a control empty vector using Effectene® (Qiagen). Culture supernatants were harvested, filtered and frozen in aliquots. The p24 content of the viruses was determined in ELISA kits (ZeptoMetrix). Target fresh 293T cells were infected with 0.5–1.5 ng equivalent of luciferase reporter viruses and cultured for 48 h. Infected cells were lysed, and each lysate was assayed for luciferase activity as previously described^10^. Values are presented as the relative infectivity relative to the value of the virus without the expression of APOBECs. The encapsidation of APOBEC proteins into HIV-1 virions was detected as described previously^10^. In brief, virus-containing supernatants were pelleted through a 20% sucrose cushion and the viral pellets were solubilized in 2 × SDS sample buffer. Then, equivalent p24 content of each solubilized virions was subjected to western blotting as described above.

### Mutation analysis of HIV-1 proviral DNA and genomic RNA by 3D-PCR

Editing of HIV-1 cDNA and genomic RNA by chimeras was verified as previously described with minor modifications^10^. To assess DNA deamination in proviral DNAs, 293T cells were infected with VSV-G pseudotyped HIV-1 as described above. 48h later, the HIV-1-infected cells were harvested and total DNAs were isolated with the QIAamp DNA Blood Mini Kit (Qiagen) followed by digestion with *Dpn* I. A 997 bp amplicon of the *tat/rev* region was amplified from the proviral DNAs with an outer primer set (Table S1). Then, the nested PCR reaction was performed at different denaturation temperatures (98 or 82 °C) to generate a 530 bp amplicon with an inner primer set in the *tat/rev* region (Table S1). The amplified products were cloned into pCR-Blunt vector and sequenced. In order to verify RNA editing activity, viral genomic RNAs in the cell-free virions were purified using the QIAamp viral RNA Mini Kit (Qiagen). Then, the isolated viral genomic RNAs were converted to cDNA by the High Capacity cDNA Archive kit (Applied Biosystems) and treated with DNase I. The viral cDNA were used as a template to amplify the *tat/rev* region at different denaturation temperatures (98 or 84 °C) as described above, and the amplified products were cloned and analyzed by sequencing.

### Statistical analysis

Data were analyzed by one-way ANOVA using GraphPad Prism 5, and *p*-values (Fisher’s LSD test) were calculated. Statistically significant correlation was determined by Pearson product-moment correlation (*r*_*p*_).

## Additional Information

**How to cite this article**: Ikeda, T. *et al.* Creation of chimeric human/rabbit APOBEC1 with HIV-1 restriction and DNA mutation activities. *Sci. Rep.* **6**, 19035; doi: 10.1038/srep19035 (2016).

## Supplementary Material

Supplementary Information

## Figures and Tables

**Table 1:** Summary of assay results.

	HIV-1 infectivity^1^	Cytoplasmic localization (%)^2^	Dimerization^3^	Incorporation efficiency^4^	DNA editing activity^5^	3D-PCR	
						Proviral DNA	Genomic RNA
Human A1	0.66 ± 0.07	24 ± 11	+	1.00 ± 0.17	2.1 ± 0.7	−	−
Rabbit A1	0.007 ± 0.001	75 ± 6	+	7.37 ± 1.63	72.8 ± 9.6	+	+
HR1	0.02 ± 0.001	51 ± 6	+	4.39 ± 1.34	8.4 ± 1.8	+	+
HR2	0.65 ± 0.05	48 ± 9	+	1.64 ± 0.36	80.4 ± 3.7	−	−
HR3	0.47 ± 0.05	36 ± 10	+	4.86 ± 2.43	22.5 ± 2.6	+	+
HR4	0.78 ± 0.05	33 ± 10	+	1.50 ± 0.29	5.1 ± 0.9	−	−
HR5	0.80 ± 0.02	43 ± 11	+	2.57 ± 0.48	9.5 ± 2.0	−	−
HR6	0.74 ± 0.04	30 ± 10	+	1.49 ± 0.16	6.4 ± 0.9	−	−
HR7	0.63 ± 0.08	35 ± 9	+	1.70 ± 0.23	9.8 ± 1.8	−	−
HR8	0.29 ± 0.03	38 ± 12	+	3.13 ± 0.70	23.7 ± 1.9	+	+
HR9	0.58 ± 0.04	26 ± 10	+	3.18 ± 1.12	21.4 ± 2.3	−	−
HR10	0.28 ± 0.02	51 ± 11	+	5.25 ± 2.05	30.3 ± 4.1	+	+
HR11	0.77 ± 0.04	26 ± 13	+	1.20 ± 0.34	6.2 ± 1.6	−	−
HR12	0.77 ± 0.09	34 ± 7	+	0.73 ± 0.24	2.6 ± 0.6	−	−
HR13	0.39 ± 0.03	41 ± 11	+	5.15 ± 0.30	68.9 ± 7.2	+	+
HR14	0.46 ± 0.06	48 ± 7	+	2.24±0.63	101.7±6.7	+	+

The anti-HIV-1 activity, cytoplasmic localization, dimerization ability, incorporation efficiency into HIV-1 virions, intrinsic DNA editing assay, and deamination activity against the proviral DNA and viral genomic are shown for human and rabbit A1s and each chimeras (HR1-14).

^1^Anti-HIV-1 activities were shown as relative value to mock infection using single-round luciferase assay (±SD).

^2^The percentage of cytoplasmic localization (±SD) of each chimera was calculated by subtracting fluorescence signal in the nucleus from total fluorescence intensity (n=30).

^3^Dimerization ability of each chimera was evaluated by immunoprecipitation.

^4^Incorporation efficiency was determined by measuring the band intensity of HA-tagged A1s or chimeras to p24 in Western blot. The value (±SD) shown is the relative intensity to that of human A1.

^5^Intrinsic DNA editing activity was measured by an *E. coli*-based system. The activity (±SD) was shown as rifampicin resistant colonies per 10^8^ viable cells.
