# Supplementary material for: Creation of chimeric human/rabbit APOBEC1 with HIV-1 restriction and DNA mutation activities
Source: Sci Rep. 2016 Jan 7;6:19035. doi: 10.1038/srep19035 (PMC4704027; doi:10.1038/srep19035)
Supplement: Supplementary Information [file srep19035-s1.doc]

**Supplementary Online Data**

# Creation of chimeric human/rabbit APOBEC1 with HIV-1 restriction and DNA mutation activities

# Terumasa Ikeda^1,§^,*, Eugene Boon Beng Ong^2^, Nobumoto Watanabe^2,3^, Nobuo Sakaguchi^4^, Kazuhiko Maeda^4,5^,* and Atsushi Koito^1^

# ^1^Department of Retrovirology and Self-Defense, Faculty of Life Sciences, Kumamoto University, Kumamoto 860-8556, Japan

# ^2^Institute for Research in Molecular Medicine, Universiti Sains Malaysia, 11800 Penang, Malaysia

# ^3^Bio-Active Compounds Discovery Research Unit, Chemical Biology Research Group, RIKEN Center for Sustainable Resource Science, 2-1, Hirosawa, Wako, Saitama 351-0198, Japan

^4^World Premier International Research Center Initiative, Immunology Frontier Research Center, Osaka University, 3-1 Yamada-oka, Suita 565-0871, Japan

^5^Laboratory of Host Defense, Research Institute for Microbial Diseases, Osaka University, 3-1 Yamada-oka, Suita 565-0871, Japan

^§^ Current address: Department of Biochemistry, Molecular Biology and Biophysics, Institute of Molecular Virology, University of Minnesota, Minneapolis, Minnesota 55455, USA.

* Correspondence may be addressed to either of these authors (E-mails: tikeda@umn.edu or kazmaeda@biken.osaka-u.ac.jp).

**Figure S1. Homodimerization of the A1s and chimeras.** Immunoblots of HA-tagged APOBEC proteins after immunoprecipitation. To test the RNA dependence of dimerization, the cell lysates were treated with RNase A before immunoprecipitation. Expression of each APOBEC protein in the transfected 293T cells was verified with Immunoblotting for each of the HA- or FLAG-tagged APOBEC proteins, as shown below. Data from one experiment representative of three independent experiments are shown.


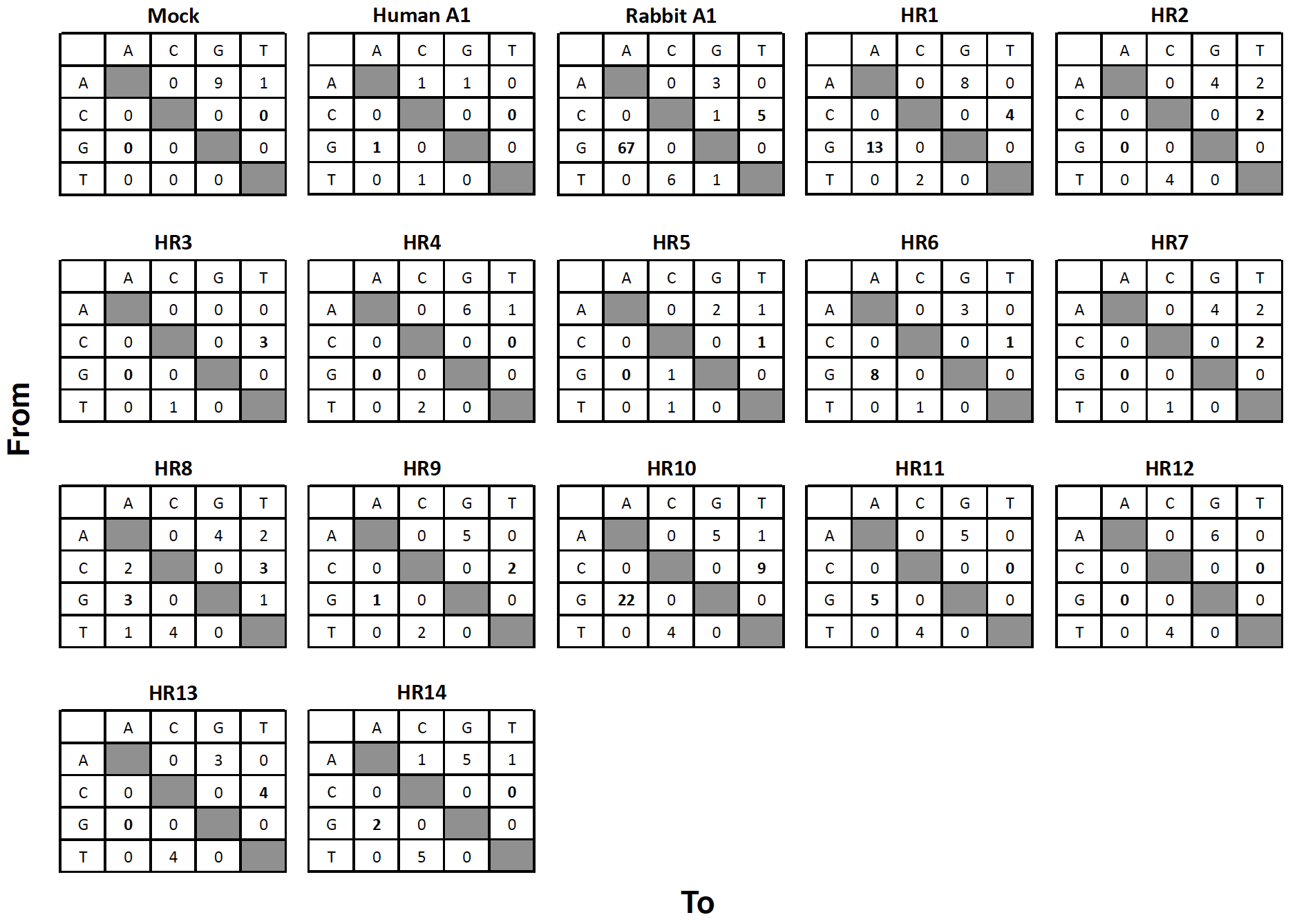


**Figure S2.** **Mutation matrices for *tat/env* genes in the proviral DNA of Vif-proficient NL-luc virus obtained from PCR amplification at 98 °C.** The isolated DNA was amplified using primer sets specific for *tat/env* region of Vif-proficient NL-luc virus at the denaturation temperature of 98 °C and a total of 12 clones were sequenced for each sample (6,384 bp). These boxes show differences between the nucleotide sequence in the Vif-proficient NL-luc virus and the observed sequence in the absence or in the presence of A1 proteins.


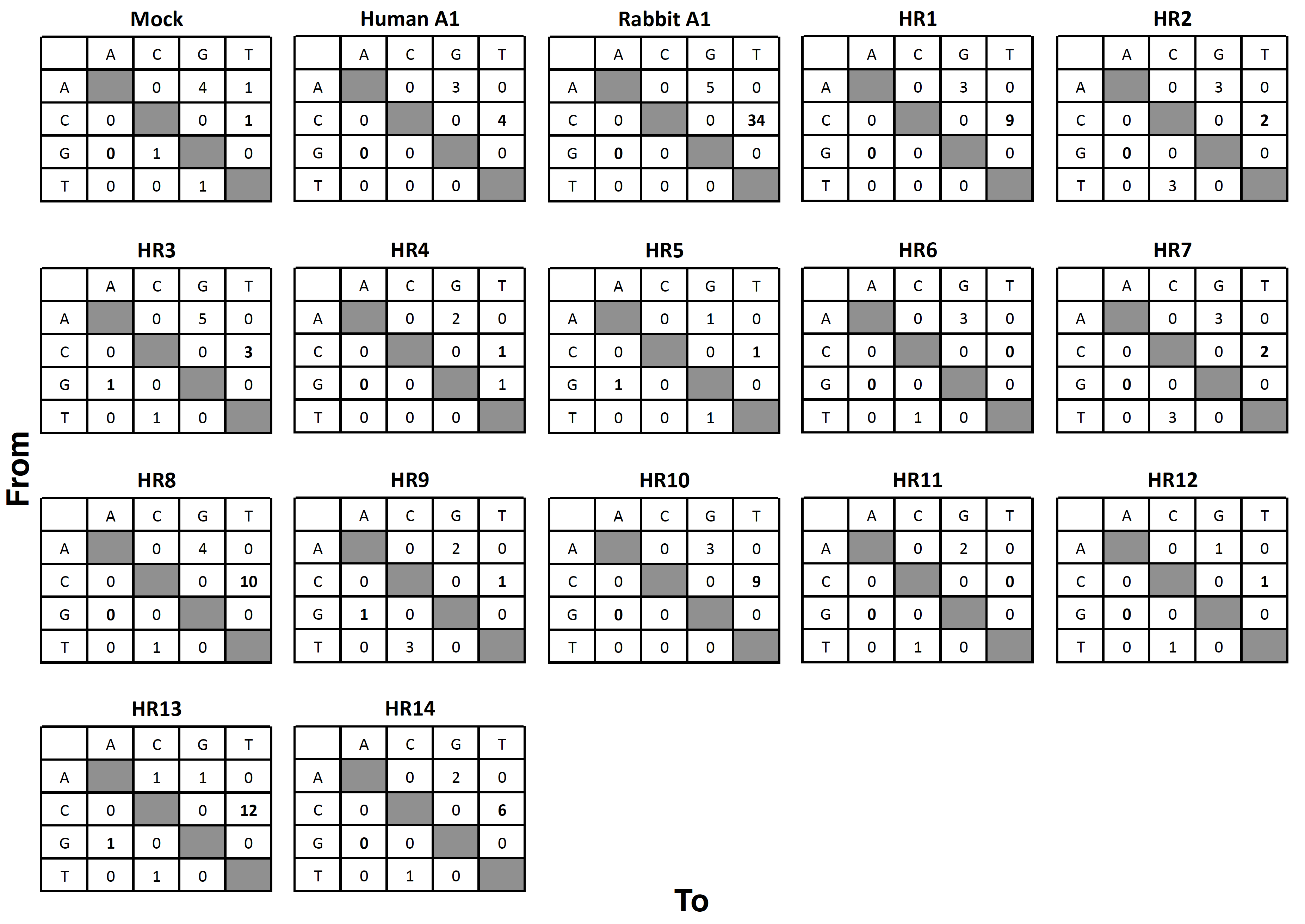


**Figure S3.** **Mutation matrices for *tat/env* genes in the genomic RNA of Vif-proficient NL-luc virus obtained from PCR amplification at 98 °C.** The isolated viral RNA was amplified using primer sets specific for *tat/env* region of Vif-proficient NL-luc virus at the denaturation temperature of 98 °C and a total of 12 clones were sequenced for each sample (6,384 bp). These boxes show differences between the nucleotide sequence in the Vif-proficient NL-luc virus and the observed sequence in the absence or in the presence of A1 proteins.

**Figure S4. Homology models of human A1 (light blue) and rabbit A1 (wheat) show structural conservation relative to A3G-CTD (light pink).** The ribbon schematics show the core five hydrophobic β-sheets (β1–β5) surrounded by six α-helices (α1–α6) arranged in the characteristic APOBEC motif. The active-core Zn^2+^-binding site is shown in green and red. Homology models were built with SWISS-MODEL and the image was generated with PyMol. This figure corresponds to Fig. 7.


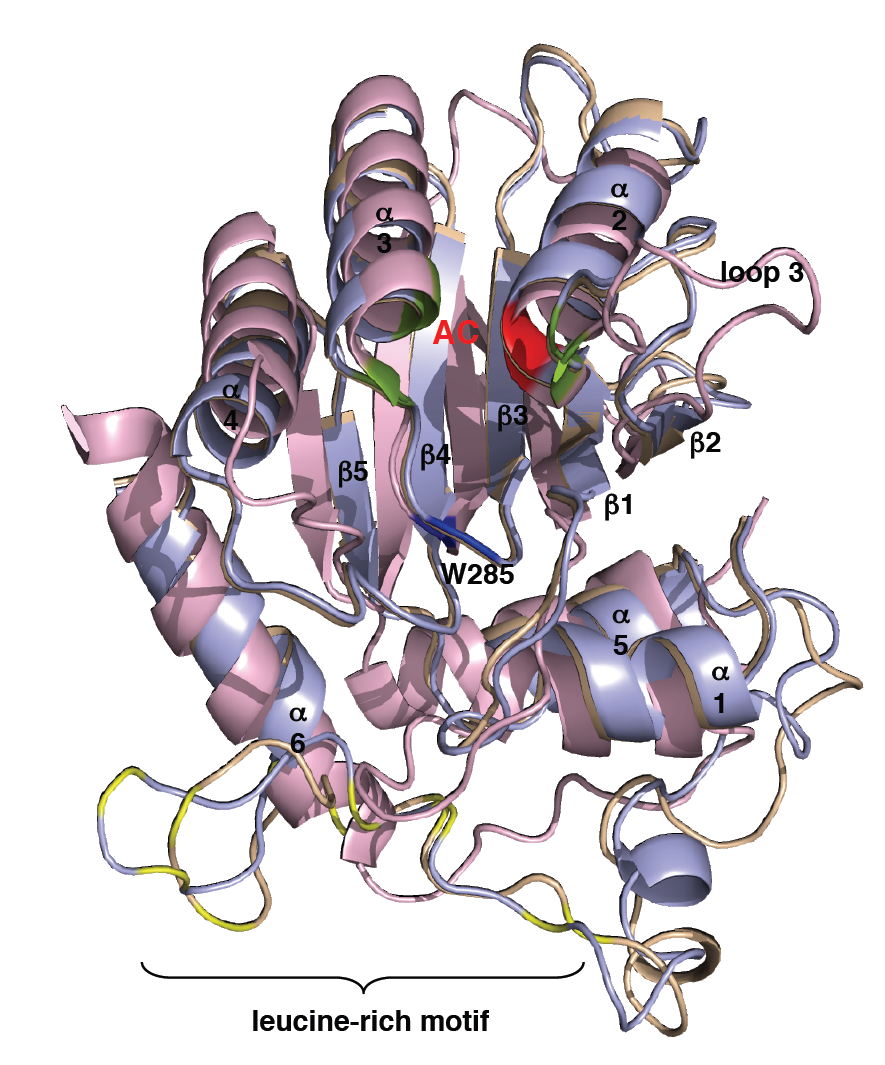


| **Table S1. Oligonucleotide sequences used in this study.** | | |
| --- | --- | --- |
| **Names** | | **Sequences (5’ to 3’)** |
| Chimera 1 | | |
| 5’ fragment | *Eco* RV-hA1 27F | GATATCAGAGCACCATGACTTCTGAGAAAGGTCC |
|  | hA1 148R | CCCCACTTGATTTCGTAGAACAGACAGGC |
| 3’ fragment | hA1 117F | GAGGCCTGTCTGCTCTACGAAATCAAGTGG |
|  | rab A1 740R-HA-*Not* I | GCGGCCGCTCAAGCGTAATCTGGAACATCGTATGGGTATCTCCAAGGCACAGAAGGTTGTAACAA |
| Chimera 2 | | |
| 5’ fragment | *Eco* RV-hA1 27F (described above) | |
|  | hA1 279R | ACAAGAACCAGGTGATGGAGCAGC |
| 3’ fragment | hA1 255F | GCTGCTCCATCACCTGGTTCTTGT |
|  | rab A1 740R-HA-*Not* I (described above) | |
| Chimera 3 | | |
| 5’ fragment | *Eco* RV-hA1 27F (described above) | |
|  | hA1 493R | GGGTAGTTGACAAAATTCCTCCAGCAG |
| 3’ fragment | hA1 467F | ACTGCTGGAGGAATTTTGTCAACTACCC |
|  | rab A1 740R-HA-*Not* I (described above) | |
| Chimera 4 | | |
| 5’ fragment | *Eco* RV-hA1 27F (described above) | |
|  | hA1 608R | TCTTCTTGAAATCTTTAAACAGGGTGG |
| 3’ fragment | hA1 582F | CCACCCTGTTTAAAGATTTCAAGAAGA |
|  | rab A1 740R-HA-*Not* I (described above) | |
| Chimera 5 | | |
| 5’ fragment | *Eco* RV-rab A1 55F | GATATCCAGAGTCAGACACCATGGCTTCCG |
|  | rab A1 150R | ACTTGATTTCGTAGAGCAGGCAGGCCTC |
| 3’ fragment | rab A1 123F | GAGGCCTGCCTGCTCTACGAAATCAAGT |
|  | hA1 742R-HA-*Not* I | GCGGCCGCTCAAGCGTAATCTGGAACATCGTATGGGTATCTCCAAGCCAC |
| Chimera 6 | | |
| 5’ fragment | *Eco* RV-rab A1 55F (described above) | |
|  | hA1 279R | ACAAGAACCAGGTGATGGAGCAGC |
| 3’ fragment | hA1 255F | GCTGCTCCATCACCTGGTTCTTGT |
|  | hA1 742R-HA-*Not* I (described above) | |
| Chimera 7 | | |
| 5’ fragment | *Eco* RV-rab A1 55F (described above) | |
|  | rab A1 498R | GGTAGTTGACAAAATTCTCCCAGCAGT |
| 3’ fragment | rab A1 472F | ACTGCTGGGAGAATTTTGTCAACTACC |
|  | hA1 742R-HA-*Not* I (described above) | |
| Chimera 8 | | |
| 5’ fragment | *Eco* RV-rab A1 55F (described above) | |
|  | hA1 608R | TCTTCTTGAAATCTTTAAACAGGGTGG |
| 3’ fragment | hA1 582F | CCACCCTGTTTAAAGATTTCAAGAAGA |
|  | hA1 742R-HA-*Not* I (described above) | |
| Chimera 9 | | |
| 5’ fragment | *Eco* RV-hA1 27F (described above) | |
|  | hA1 499R | GGGTAGTTGACAAAATTCCTCCAGCAG |
| 3’ fragment | hA1 473F | CTGCTGGAGGAATTTTGTCAACTACCC |
|  | hA1 742R-HA-*Not* I (described above) | |
| Chimera 10 | | |
| 5’ fragment | *Eco* RV-rab A1 55F (described above) | |
|  | rab A1 498R | GGTAGTTGACAAAATTCTCCCAGCAG |
| 3’ fragment | rab A1 473F | CTGCTGGGAGAATTTTGTCAACTACC |
|  | rab A1 740R-HA-*Not* I (described above) | |
| Chimera 11 | | |
| 5’ fragment | *Eco* RV-hA1 27F (described above) | |
|  | rab A1 498R | GGTAGTTGACAAAATTCTCCCAGCAG |
| 3’ fragment | rab A1 473F | CTGCTGGGAGAATTTTGTCAACTACC |
|  | hA1 742R-HA-*Not* I (described above) | |
| Chimera 12 | | |
| 5’ fragment | *Eco* RV-hA1 27F (described above) | |
|  | hA1 361R | AGAGTCACACCAGGGTGCCGACTC |
| 3’ fragment | hA1 327F | GAGTCGGCACCCTGGTGTGACTCT |
|  | hA1 742R-HA-*Not* I (described above) | |
| Chimera 13 | | |
| 5’ fragment | *Eco* RV-rab A1 55F (described above) | |
|  | hA1 499R | GGGTAGTTGACAAAATTCCTCCAGCAG |
| 3’ fragment | hA1 473F | CTGCTGGAGGAATTTTGTCAACTACCC |
|  | rab A1 740R-HA-*Not* I (described above) | |
| Chimera 14 | | |
| 5’ fragment | *Eco* RV-rab A1 55F (described above) | |
|  | rab A1 357R | AGAGTCACACCCGGGTGTTGACTC |
| 3’ fragment | rab A1 333F | GAGTCAACACCCGGGTGTGACTCT |
|  | rab A1 740R-HA-*Not* I (described above) | |
| HIV-1 *tat*/*env* sequence | 5889F | TTGTACCAATTGCTATTGTAAAAAGTGTTGCTT |
|  | 6062F | TGCAACCTATAATAGTAGCAATAGTAGCATTAGTAGTAGCAA |
|  | 6591R | GGGGTTAATTTTACACATGGCTTTAGGC |
|  | 6885R | AACCAGCCGGGGCACAATA |
| Bacterial mutator assay | *Xho* I-hA1 | CTCGAGATGACTTCTGAGAAAGGTCCTTCAACCG |
|  | *Xho* I-rab A1 | CTCGAGATGGCTTCCGAGAAAGGTCCTTCAAA |
|  | HA-*Pst* I | CTGCAGTCAAGCGTAATCTGGAACATCGTATGGGTA |
| FLAG-tagged APOBECs | *Eco* RV-hA3G | GATATCATGAAGCCTCACTTCAGAAACACAGTGGAG |
|  | hA3G-FLAG-*Not* I | GCGGCCGCTCACTTGTCGTCATCGTCTTTGTAGTCGTTTTCCTGATTCTGGAGAATGGCC |
|  | hA1 682R-FLAG-*Not* I | GCGGCCGCTCACTTGTCGTCATCGTCTTTGTAGTCTCTCCAAGCCACAGAAGGATGTATCAG |
|  | rab A1 682R-FLAG-*Not* I | GCGGCCGCTCACTTGTCGTCATCGTCTTTGTAGTCTCTCCAAGGCACAGAAGGTTGTAACAA |
| Restriction enzyme sites were shown in underline. | | |
